# The tumor-associated fibrotic reactions in microenvironment aggravate glioma chemoresistance

**DOI:** 10.3389/fonc.2024.1388700

**Published:** 2024-05-28

**Authors:** Jiaqi Xu, Ji Zhang, Wubing Chen, Xiangrong Ni

**Affiliations:** ^1^ The Second Clinical Medical School, Zhujiang Hospital, Southern Medical University, Guangzhou, China; ^2^ Department of Neurosurgery, Sun Yat-sen University Cancer Center, Guangzhou, China; ^3^ Department of Radiology, Wuxi Fifth People’s Hospital, Jiangnan University, Wuxi, China; ^4^ Translational Medicine Research Center, Zhujiang Hospital, Southern Medical University, Guangzhou, China; ^5^ Department of Plastic Surgery, Zhujiang Hospital, Southern Medical University, Guangzhou, China

**Keywords:** chemoresistance, tumor-associated fibrotic reaction, glioma, antifibrosis therapy, tumor microenvironment (TME), cancer-associate fibroblasts

## Abstract

Malignant gliomas are one of the most common and lethal brain tumors with poor prognosis. Most patients with glioblastoma (GBM) die within 2 years of diagnosis, even after receiving standard treatments including surgery combined with concomitant radiotherapy and chemotherapy. Temozolomide (TMZ) is the first-line chemotherapeutic agent for gliomas, but the frequent acquisition of chemoresistance generally leads to its treatment failure. Thus, it’s urgent to investigate the strategies for overcoming glioma chemoresistance. Currently, many studies have elucidated that cancer chemoresistance is not only associated with the high expression of drug-resistance genes in glioma cells but also can be induced by the alterations of the tumor microenvironment (TME). Numerous studies have explored the use of antifibrosis drugs to sensitize chemotherapy in solid tumors, and surprisingly, these preclinical and clinical attempts have exhibited promising efficacy in treating certain types of cancer. However, it remains unclear how tumor-associated fibrotic alterations in the glioma microenvironment (GME) mediate chemoresistance. Furthermore, the possible mechanisms behind this phenomenon are yet to be determined. In this review, we have summarized the molecular mechanisms by which tumor-associated fibrotic reactions drive glioma transformation from a chemosensitive to a chemoresistant state. Additionally, we have outlined antitumor drugs with antifibrosis functions, suggesting that antifibrosis strategies may be effective in overcoming glioma chemoresistance through TME normalization.

## 1 Introduction

Gliomas are stratified into grades 1 through 4 according to the World Health Organization’s tiered grading system, and grade 4 is the most prevalent and virulent subtype, also known as glioblastoma. GBM, an unyielding primary cerebral malignancy, has a grim prognosis with a 5-year survival rate of less than 10% (1–3), despite standard therapies including the maximal tumor excision, combined with concomitant radiotherapy and temozolomide chemotherapy. So far, TMZ is the first-line chemotherapy drug for glioma. However, due to the frequent occurrence of TMZ resistance after chemotherapy, glioma is recalcitrant and refractory. To increase the prognosis of GBM patients, it’s important to summarize the potential mechanisms of glioma chemoresistance and find useful strategies to overcome TMZ resistance.

Gliomas are characterized as easily chemoresistant intracranial malignancy through demethylation of O(6)-methylguanine-DNA methyltransferase (MGMT) promoter, overexpression of cell membrane glycoprotein, and the augmentation of stemness-associated molecules (4–7). Moreover, the chemoresistance could not only be developed by the cellular alterations in cancer cells but also, in part, be modulated by the specific TME (8). Many researchers recently have focused on the chemoresistance promoted by the GME and are increasingly aware of the significance of overcoming chemoresistance by normalizing GME. The nonneoplastic immune cells and stromal components foster an immunosuppressive GME under the interaction of glioma-secreted cytokines (9, 10). The prominent nonneoplastic stromal cells in gliomas consist of endothelial cells, microglia, and tumor-associated macrophages (TAMs), etc. (2, 11–13). In solid tumor stroma, cancer-associated fibroblasts (CAFs) secrete a lot of collagen after stimulation (14), and subsequently increase the stiffness of the tumor matrix which in turn enhances the proliferation, invasiveness, and stemness as well as chemoresistance of glioma cells. Different from others’ attention on the glioma chemoresistance increased by the alterations of glioma cells themselves, in this review, we summarize the relationship between the chemoresistance and glioma-associated fibrotic reactions. Several investigators have attempted to enhance TMZ chemotherapy efficacy with reasonable combinations of some clinically approved conventional drugs (15), and among these drugs, the increased chemotherapy efficacy by some agents with antifibrosis function draws our attention. However, it is so little known why the antifibrosis medication is effective for solid tumors and how glioma-associated fibrotic reactions in GME specifically contribute to TMZ chemoresistance and poor prognosis for glioma patients. In this review, we thus explore the mechanism of the occurrence and development of tumor-associated fibrotic phenomena in GME and sum up the antifibrosis strategies for sensitizing chemotherapy, hoping to provide novel insights for glioma research and treatment.

## 2 The formation of tumor-associated fibrotic reactions in the tumor microenvironment

During the malignant progression of tumor cells, changes in the tumor stroma also take place including alterations of extracellular matrix (ECM) components, stroma stiffness, excessive vascularization, hypoxia, and paracrine cytokine secretion. As a principal non-cellular component, the ECM plays a crucial role in driving tumor malignancy by providing cells with architectural and mechanical supports, regulating nutrient supply, as well as engaging in multiple cellular processes as a reservoir of diverse cytokine regulators (16–19). The ECM components would transform into a specific status that can stimulate the growth of cancer cells and tumor-associated cells. As cancer occurs and develops, malignant and stromal cells can deposit, break down, and remodel the ECM through the production of multiple ECM proteins, including collagens, fibronectins, laminins, and proteolytic enzymes, which can stimulate the growth of cancer cells and tumor-associated cells (20). In addition, alterations in the biophysical properties of the ECM, such as stiffness, density, rigidity, tension, and protein deposition, are recognized as hallmarks of tumor stromal fibrosis (21, 22). In the TME, CAFs are one of the most critical stromal cell types functioning as the architects of matrix remodeling, which provides the “soil” for tumor survival (23). CAFs could be identified with molecular markers such as αSMA, FAP-1, desmin, podoplanin, NG2 (CSPG4), and PDGFR-α/β (24). These CAFs could secrete substantial quantities of ECM components after being activated and could mediate the malignant progression of tumors through the promotion of stromal inflammation and fibrosis (24, 25). Cancer cells exhibit multiple features of cancer progression, including the recruitment of various stromal cells to form the TME (26), which encompasses different functional subtypes of stromal cells and matrix polymers (27). Among these cells, CAFs promote the formation of a dense and rigid fibrotic microenvironment by large amounts of ECM proteins and cytokines secretions (28). In TME, tumor-associated fibrosis is the result of excessive accumulation of collagen, fibronectin, laminin, tendon protein, etc. (29, 30). Among these ECM components, the most abundant one is collagen protein which constitutes the main rigid structures of tumor stroma. There are more than 28 types of collagens which are categorized into four subtypes: fibril-forming collagens (I, II, III, V, XI, XXVI, XXVII), fibril-associated collagens with interrupted triple helices (FACITs: IX, XII, XIV, XVI, XIX, XX, XXI, XXII, XXIV), network-forming collagens (IV, VIII, X), and membrane-anchored collagens (MACITs: XIII, XVII, XXIII, XXV) (31). Among these collagens, types I, III, and V collagen are predominantly secreted by CAFs, while type IV collagen is mainly produced by epithelial and endothelial cells. It is worth noting that, under certain conditions, tumor cells and TAMs can also synthesize collagen (32). Collectively, tumor-associated fibrotic reactions are induced by the interactions between tumor cells and stromal cells.

In glioma, radiotherapy and cytotoxic chemotherapy can induce epithelial-mesenchymal transition (EMT) and upregulate the transforming growth factor-β (TGF-β) signal, which is the key signaling pathway to fibrosis initiation (33). EMT is associated with increased expression of TGF-β, collagen, fibronectin, α-SMA, and S100A4, suggesting that these molecular mechanisms could be involved in inducing stromal fibrotic reactions in glioma. Some researchers have proposed a “repurposing” strategy for treating GBM by using clinically approved conventional drugs to inhibit EMT. For instance, Kast et al. summarized six clinically approved drugs including fenofibrate, quetiapine, lithium, nifedipine, itraconazole, and metformin (33). These drugs are being explored as adjunctive agents to enhance chemosensitivity in tumor therapy. Given the heterogeneity of GBM, further research is needed to determine which molecular subtypes may benefit from these non-antitumor drugs.

## 3 Fibrotic components in extracellular matrix facilitate the malignant progression of glioma

**Figure 1:**
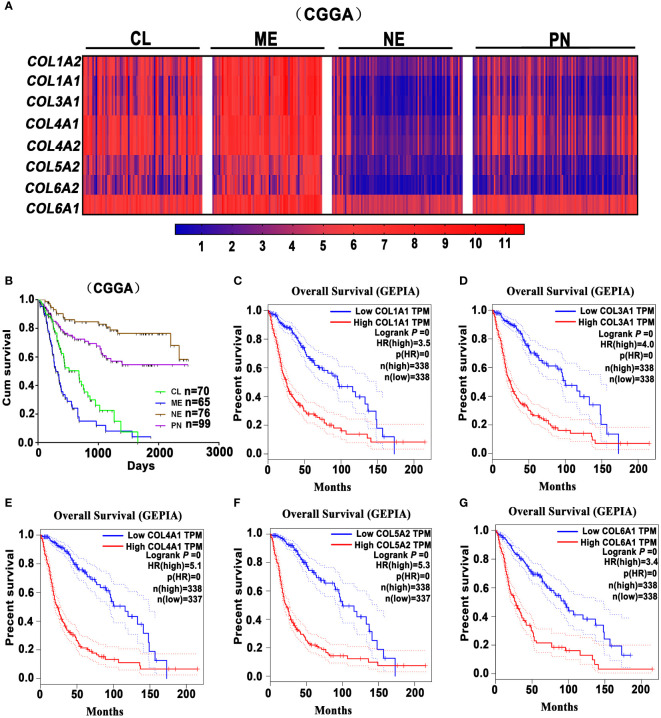
Glioma-associated fibrosis predicts poor prognosis of glioma patients. **(A)** The expression analysis of fibrosis-related marker genes *COL1A2*, *COL1A1*, *COL3A1*, *COL4A1*, *COL4A2*, *COL5A2*, *COL6A2*, *COL6A1* in the Proneural (PN), Classical (CL), Mesenchymal (ME), and Neural (NE) GBM subtypes in the CGGA dataset. **(B)** Kaplan-Meier survival curves indicate that ME and CL GBM subtypes predict a poor prognosis for glioma patients in the CGGA dataset. **(C–G)** Kaplan-Meier survival curves indicate that the high expression of collagen-related genes (*COL1A1*, *COL3A1*, *COL4A1*, *COL5A2*, and *COL6A1*) correlates with a poor prognosis for glioma patients in the GEPIA dataset. (GEPIA dataset includes TCGA LGG and GBM dataset.).

The ECM in the normal brain tissues predominantly consists of hyaluronic acid, proteoglycans, and laminin, but very little collagen. However, in GBM, there is a substantial presence of collagen proteins, laminin, and fibronectin, primarily distributed in the vascular basement membrane in tumor tissue (34). Recent reports highlighted the role of collagen in enhancing GBM cell stemness and promoting EMT and invasion (35, 36). Some researchers found that type-I collagen, the main component of tumor-associated fibrosis, could be used to promote the formation of an invasive, tight GBM spheroid structure when the collagen concentrations increase to some extent (37). Huijbers et al. conducted histological analyses on 90 GBM cases, revealing a significant abundance of collagen proteins within the ECM of GBM (38). They also observed that the collagen receptors Endo180 are overexpressed on the surface of GBM cells. It is worth noting that Endo180 expression is particularly pronounced in stromal-rich high-grade gliomas (39), and its regulation is linked to the TGF-β signaling pathway (40). Additionally, the interactions between Endo180 and collagen significantly potentiates GBM invasion (38). In GBM, collagen XVI induces tumor invasion by modulating the activation pattern of integrin β1, possibly impacting the interactions between glioma cells and the stroma to further enhance the invasive phenotype (41). Furthermore, in order to confirm the association between tumor-associated fibrotic reactions and glioma prognosis, we analyzed the Chinese Glioma Genome Atlas (CGGA) (http://www.cgga.org.cn/) and Gene Expression Profiling Interactive Analysis (GEPIA) (http://gepia.cancer-pku.cn/index.html) databases. According to the CGGA dataset, fibrosis-related marker genes (*COL1A2*, *COL1A1*, *COL3A1*, *COL4A1*, *COL4A2*, *COL5A2*, *COL6A2*, *COL6A1*) are highly expressed in the mesenchymal (ME) and classical (CL) GBM subtypes, which are associated with shorter overall survival (**Figures 1A, B**). Meanwhile, analysis of the GEPIA dataset reveals that the high expressions of collagen-related genes (*COL1A1, COL3A1, COL4A1, COL5A2*, and *COL6A1*.) are associated with poor prognosis of glioma patients (**Figures 1C–G**). Collectively, it suggests that heavier fibrotic reactions play a critical role in glioma progression and predict a poor prognosis for glioma patients.

Knocking out the collagen XVI gene in GBM U87MG cells resulted in a significant decrease in invasive capabilities compared to the control group (42). Experimental evidence has also shown that the GBM cell compaction promotes the expression of more collagen proteins and vascular endothelial growth factors in GBM, notably elevating the mRNA and protein levels of collagen types VI and IV, as well as the collagen crosslinker named lysyl oxidase (LOX). Notably, β-aminopropionitrile (BAPN), a collagen inhibitor, significantly inhibits collagen crosslinking in the ECM components of GBM by specifically targeting and suppressing LOX. Studies concurrently demonstrated that LOX expression controls the malignant progression of GBM. In an *in situ* GBM mouse model, treatment with BAPN markedly inhibited intracranial tumor growth by suppressing LOX activities (34). In conclusion, these findings underscore the pivotal role of tumor-associated fibrotic components in fostering the malignant progression of glioma, shedding light on the potential therapeutic effect of antifibrosis medications for controlling this devastating disease.

## 4 The chemoresistance can be enhanced by glioma-associated fibrotic reactions

There are two aspects explaining the mechanism by which glioma-associated fibrotic reactions induce chemoresistance. Firstly, after treated with cytotoxic drugs in solid tumors, CAFs and mesenchymal stem cells (MSCs) are recruited and increased in ECM, along with the accumulation of cytokines and other secreting factors. This fortifies the tumor “stemness”, thereby leading to chemoresistance (43). Secondly, the increased stiffness of tumor tissues with heavily fibrotic ECM components hinders the delivery of chemotherapeutic agents to tumor cells. This limits the penetration of drugs into tumor cells, thereby impairing chemotherapy efficacy (29).

### 4.1 Tumor-associated fibrotic reactions promote the stemness of glioma cells

The increased resistance of glioma stem cells (GSCs) to chemotherapeutic agents, which contribute to glioma refractoriness and recurrence, has been extensively documented (44, 45). GSCs are known to cause TMZ resistance through the upregulation of MGMT protein levels (46, 47). In TME, Oleynikova et al. found that the CAFs form the niche for tumor stem cells and these compartments surrounding tumor cells facilitated chemotherapy resistance. In agreement, CAFs may promote the stemness of cancer cells by establishing a survival niche to sustain cancer stem cells (CSCs) and protecting them from chemotherapy-induced cell death, hence, facilitating chemotherapy resistance (25, 28, 48). While it remains unclear how glioma-associated fibrotic reactions develop and which of the molecular characteristics of gliomas is more likely to form fibrosis. It has been widely reported that fibroblast activation protein-α (FAP-α) is involved in tumor-associated fibrosis. FAP-α, typically undetectable in normal tissues, however, exhibits overexpression within glioma cells and glioma stroma (49). Its selective localization in the tissue remodeling and repairing sites enhances the invasiveness and malignant progression of solid tumors including gliomas, implicating FAP-α as a potential target for addressing tumor-associated fibrosis dysregulation (12, 50). Currently, it has been reported that PT-100 significantly reduces CAF enrichment by targeting FAP-α in the tumor stroma, which enhances chemotherapeutic efficacy and reduces drug resistance when combined with oxaliplatin for colon cancer treatment (51). Also, the bone marrow derived MSCs have been demonstrated to enhance tumor stemness after being recruited to the tumor stroma, either through direct paracrine signaling or via its transformation into CAFs (43). Jia et al. compared the gene expression profiles of glioma cells between three-dimensional culture with collagen scaffolds and the conventional two-dimensional culture, and they found that collagen scaffolds could upregulate the expression of EMT-related molecules N-cadherin and vimentin, invasion-related matrix metalloproteinases (MMPs) such as MMP1, MMP2, MMP3, and MMP7, as well as stemness-associated factors CD133, Nestin, Oct4, Sox2, c-Myc, Nanog, MSI1, MSI2 and BMI-1, etc. (35). The glioma stroma harbors a substantial population of TAMs and microglia, which secrete high levels of TGF-β. This cytokine in turn, promotes the invasiveness of CD133(+) GSCs. Moreover, the upregulated TGF-β1 levels are associated with the increased MMP9 production in GSCs (36). What’s more, high serum levels of TGF-β positively correlate with poor prognosis in GBM, hinting at its pivotal role in the maintenance of glioma stemness and malignancy (52). Currently, the strategies targeting the TGF-β signaling have exhibited promising safety and efficacy profiles for gliomas. For instance, anti-TGF-β antibodies have significantly prolonged the survival of recurrent glioma patients (53). It has been proved that after TMZ treatment, the activation of the TGF-β signaling in GBM leads to connective tissue growth factor (CTGF) overexpression, which subsequently mediates TMZ resistance by enhancing the stemness of glioma cells (54). Therefore, these findings suggest that tumor-associated fibrotic reactions play a role in promoting chemoresistance by enhancing the stemness and EMT of glioma cells.

### 4.2 Glioma-associated fibrosis reduces drug delivery efficiency

The ECM consists of a variety of structural proteins that maintain tissue structure and regulate extracellular biochemical signals, thereby modulating cellular functions (18, 55). In the process of traveling from blood vessels to tumor cells, chemotherapeutic drugs must navigate through the ECM to reach their target cells. However, drug penetration can be hindered by low pH conditions that facilitate the binding of positively charged chemotherapy drugs to negatively charged ECM components, ultimately reducing the efficiency of drug delivery to cancer cells (29). What’s more, positively charged drugs have more difficulty in crossing the hydrophobic plasma cell membranes (56, 57). As we know, only after the chemotherapeutic drugs penetrate cell membranes and reach the nucleus can they adequately exert their cytotoxic effects (29).

It is known that tissue stiffness varies across different diseases and organs; for instance, normal liver tissue exhibits a “stiffness” at 6 kPa, while the “stiffness” of fibrotic liver tissues can reach up to 12 kPa (58). As the tumor develops, the deposition of type I and IV collagen increases in the cross-linking and tumor-associated fibrosis process, leading to an increase in the “stiffness” of the ECM (59). The increased ECM stiffness corresponds to upregulated contractile and traction forces of the cell cytoskeleton as cells attempt to balance extracellular tension (29). Intercellular mechanotransduction is the process of converting external mechanical stimuli into intracellular biochemical signals. Changes in ECM stiffness are sensed by local junctions between cells, and these junctions are protein complexes containing mechanosensitive protein molecules such as talin and integrins (60). Due to the tension between intracellular contractile forces and extracellular stiffness, talin unfolds in response to the forces, resulting in the exposure of hidden intracellular binding sites that allow effector proteins to bind (61). What’s more, focal adhesion kinase (FAK) is another element that can be activated by external rigidity, and this kinase activity can be utilized to initiate intracellular signaling pathways such as Yes-associated protein (YAP) nuclear localization (62). As mentioned before, the heightened tumor stiffness is closely associated with ECM compositions such as MMPs, hyaluronic acid, and abundant collagens and their cross-linking (18). During the progression of tumors, the accumulation of mechanical pressure can compress tumor blood vessels and lymphatic vessels, leading to reduced perfusion, hypoxia, and elevated interstitial pressure within tumor tissues, thus reducing chemotherapy efficacy (63, 64). Therefore, strategies aiming at reducing mechanical stress in glioma, such as tumor decompression therapy (65), can relieve vascular compression within tumors, enhance tissue perfusion, and improve the transport of chemotherapeutic drugs into tumor cells.

It is important to recognize that chemoresistance, in part, is modulated by collagen and hyaluronic acid in the TME (66) and the strategies specifically targeting these components may be useful tumor decompression therapies. Surprisingly, repurposing those conventionally approved drugs with antifibrosis function can indeed inhibit tumor growth by normalizing TME with the downregulated ECM synthesis. This, in turn, reduces tumor stiffness and mechanical stress, relieves vascular and lymphatic compression, and enhances drug permeability into tumor tissues (67–69). Currently, antifibrosis drugs such as tranilast, losartan, and pirfenidone, have been used to improve chemotherapy in solid tumors (63, 70, 71), while it warrants subsequent research to confirm their efficacy in glioma. As for stroma-rich tumors, researchers concentrate on developing nanomedicines targeting CAFs to reduce tumor matrix stiffness (72, 73). These nano-delivery systems have a double effect on enhancing chemotherapy. Firstly, they reverse tumor progression, immunosuppression, or drug-resistance phenotypes by inhibiting signaling between CAFs and tumor cells, thus increasing chemosensitivity. Secondly, by weakening CAFs function, nanomedicines reduce tumor solid-phase pressure, tumor tissue fluid pressure, and ECM density, leading to increased penetration depth of antitumor drugs and improved efficiency of chemotherapeutic drug delivery.

## 5 Molecular regulatory mechanisms of glioma-associated fibrosis

MMPs are a group of zinc-dependent endopeptidases involved in the dynamic remodeling of ECM, exhibiting proteolytic activities toward ECM components such as collagen (74). In normal circumstances, the synthesis and degradation of ECM is a homeostatic process regulated by the balanced activity of MMPs. However, in tumor tissues, this homeostasis is disrupted due to the overexpressed or hyperactivated of MMPs in gliomas, such as MMP2, MMP9 MMP3, MMP13, MMP14, MMP19, MMP26, and MMP28 (75–84). After effective treatment of U87 glioma xenografts with TMZ, MMP expression is downregulated, with the downregulation of MMP2 and MMP3 associated with the inhibitory effects of TMZ on gliomas (85). In addition, MMPs can promote tumor invasion by facilitating tumor cell degradation of the surrounding matrix or by activating paracrine signaling factors through proteolytic cleavage. For instance, MMPs can lead to the secretion of large amounts of TGF-β, which subsequently promotes CAF activation, long-term fibrosis, and MMP expression and secretion (86).

TME contains numerous signaling molecules and growth factors that, upon binding to cell surface receptors, initiate intracellular signaling in cancer cells, ultimately leading to changes in gene expression. Signaling factors through this mechanism are significantly increased in tumors, for instance, growth factors such as epidermal growth factors (EGFs), fibroblast growth factors (FGFs), platelet-derived growth factors (PDGF), and hepatocyte growth factors (HGF) are abundant in TME (87). During ECM remodeling, the secretion of MMPs promotes the release of growth factors in ECM, such as TGF-β (18, 88). TGF-β exhibits a dual regulatory role in tumor cells, promoting both apoptosis and survival. It suggests that the switch from proapoptotic to prosurvival signaling in tumor cells is influenced by the TP53 gene mutation status (89) or the stiffness of the ECM (90). After the activation of CAFs by TGF-β, they play a crucial role in mediating the maintenance of the TME through paracrine signaling pathways (91). Both glioma cells and infiltrating immune cells in TME could secrete various cytokines, including TGF-β, CTGF, IL-6, and IL-10, contributing to the formation of an immunosuppressive GME (52), many of these cytokines promote chemoresistance in gliomas. Research has shown that in GBM, when treated with TMZ, the activation of the TGF-β signaling pathway leads to the overexpression of CTGF, and subsequently, CTGF increases the expression levels of glioma stem cell markers, including ALDH1, CD44, Nestin, and Nanog (54). In conclusion, the fibrotic alterations in GME are closely related to the maintenance of glioma cell stemness and the chemoresistance of glioma.

## 6 Tumor-associated fibrotic reactions contribute to immunotherapy resistance and TAMs-mediated chemoresistance in glioma

**Figure 2:**
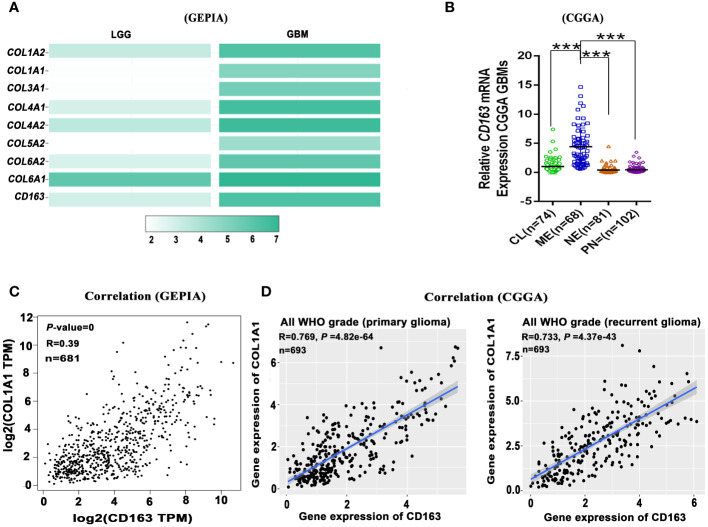
Glioma-associated fibrosis positively correlates with the expression of macrophage marker gene *CD163.* **(A)** The expression of fibrosis-related marker genes (*COL1A2*, *COL1A1*, *COL3A1*, *COL4A1*, *COL4A2*, *COL5A2*, *COL6A2*, *COL6A1*) and M2 macrophage marker gene (*CD163*) in low-grade glioma (LGG) and GBM in the GEPIA dataset. **(B)** Relative *CD163* mRNA expression levels of four GBM subtypes in the CGGA dataset. ***p < 0.001. **(C)** *CD163* expression positively correlates with *COL1A1* expression in all grade glioma (GEPIA). **(D)** *CD163* expression positively correlates with *COL1A1* expression in primary and recurrent glioma (CGGA). (GEPIA dataset includes TCGA LGG and GBM dataset.).

Nowadays, numerous glioma immunotherapies have been investigated in clinical and preclinical phases. These include immune checkpoint blockade targeting IDO, CTLA-4, and PD-L1 (92), as well as inhibitors of M2 macrophages such as CSF-1R (93, 94), PI3Kγ (8), and BAPN (11), and antibodies targeting cytokines like IL-6 (95), and CCL5 (96, 97), etc. However, the efficacy of many immunotherapy strategies for GBM remains very limited due to the absence of T lymphocytes, B lymphocytes, and NK cells, as well as the presence of the blood-brain barrier (BBB). Furthermore, during the process of tumor-associated fibrotic reactions, the stiff ECM, particularly the highly crosslinked collagen, creates hypoxic conditions in and around the TME (98) and hinders the infiltration of immune cells or immunotherapeutic agents into tumor tissues (99). Therefore, tumor-associated fibrotic reactions play a role in promoting an immunosuppressive TME, which mediates the immunotherapy resistance in solid tumors. As widely known, the mesenchymal subtype of GBM is characterized by abundant immune features (100), especially the M2 macrophages and microglias (101), suggesting that targeting macrophages could be a useful strategy for treating mesenchymal subtype GBM. Interactions between CAFs and M2 macrophages play a crucial role in the formation of tumor-associated fibrotic reactions (102). As is known, macrophages, contributing to glioma progression (11, 103), can also release significant amounts of TGF-β to initiate and accelerate fibrotic reactions. Furthermore, our investigation revealed that fibrosis-related collagens expression and M2 macrophage marker *CD163* expression may participate in glioma malignancy, and analysis from the GEPIA database shows that these collagens (*COL1A2*, *COL1A1*, *COL3A1*, *COL4A1*, *COL4A2*, *COL5A2*, *COL6A2*, *COL6A1*) and *CD163* expression are higher in GBM compared to low-grade glioma (LGG) (**Figure 2A**). The mesenchymal subtype of GBM exhibits severe glioma-associated fibrotic reactions, characterized by the most prominent collagen deposition and highest macrophage infiltration in the CGGA and GEPIA datasets (**Figures 1A**, **2B**). It also suggests that the expression level of COL1A1 positively correlates with the expression level of CD163 (the M2 macrophage marker gene) (**Figures 2C, D**). TAMs-secreted IL-11 and LOX factors promote glioma chemoresistance and progression, while PI3Kγ inhibition (8) and LOX inhibitors (11) could significantly improve TMZ efficacy in orthotopic GBM mouse models. Furthermore, IL-11 (104) and LOX (105), two crucial determinants of tissue fibrosis, are therapeutic targets against organ fibrosis. This suggests that antifibrosis strategies may enhance chemosensitivity in glioma. Therefore, glioma progression and chemoresistance are not only directly promoted by M2 macrophage-secreted cytokines (IL-10, TGF-β, IL-6, etc.) but also modulated by M2 macrophage-mediated fibrotic reactions. Collectively, it suggests that fibrotic reactions partly contribute to macrophage-mediated chemoresistance.

## 7 Antifibrosis therapies explored and tested in glioma

Researchers have explored the antifibrosis therapies in solid tumors, showing definitive sensitization effects for chemotherapy. However, in the presence of the BBB, further research is warranted to determine the efficacy of antifibrosis drugs in sensitizing glioma chemotherapy. In this context, we have summarized the information concerning the utilization of antifibrotic therapies in glioma. According to the existing classifications of antifibrosis therapies by scholars (106), we conclude and discuss antifibrosis therapies in glioma as follows.

### 7.1 Targeting ECM and ECM modulators

As previously discussed, ECM components could be transformed into pro-tumor phenotypes during tumor progression. This transformation presents numerous viable antifibrosis targets for improving chemotherapy by reducing ECM stiffness through downregulated ECM deposition and collagen-modifying enzymes.

Collagen is a prominent component of the ECM, and inhibiting collagen cross-linking has demonstrated significant efficacy in orthotopic GBM mouse models (34). LOX, a kind of collagen cross-linking enzyme, is significantly upregulated during glioma progression due to “cell compaction”. Studies indicate that BAPN, a LOX inhibitor, can effectively inhibit the growth of intracranial *PTEN*-null GBM mouse models (34, 107). This suggests that BAPN can be a promising strategy for inhibiting glioma growth, possibly by negatively modulating tumor-associated fibrosis. Similarly, another enzyme involved in the process of collagen cross-linking, procollagen-lysine 2-oxoglutarate 5-dioxygenase 2 (PLOD2), has also been tested in GBM treatment. Elevated PLOD2 expression is significantly associated with GBM proliferation, invasion, metastasis, and poor overall survival (108–110). PLOD2 participates in the formation of tumor-associated fibrosis through promoting EMT transition (111), possibly via FAK (108), and PI3K-Akt (111) signaling pathways. Both *in vivo* and *in vitro* studies have demonstrated that PLOD2 knockdown inhibits the proliferation, invasion, and anchorage-independent growth of GBM (108, 110, 111). Minoxidil, a confirmed PLOD2 inhibitor (112), could suppress tumor metastasis, in part, by reversing collagen cross-linking in ECM (113, 114). Moreover, studies have found that minoxidil cloud increases the antitumor drug permeability of the blood-brain tumor barrier, resulting in improved and selective delivery to brain tumors, including GBM (115, 116). In conclusion, PLOD2 could serve as a viable target against glioma, possibly by normalizing GME with its antifibrosis function, and PLOD2 inhibitors like Minoxidil may offer potential benefits for glioma patients.

### 7.2 Targeting TGF-β signaling pathway

The TGF-β signaling pathway is recognized as the key signal that mediates tissue fibrosis processes and contributes to cancer progression (117, 118). It has been extensively explored whether repurposing antifibrosis drugs can increase chemotherapy sensitivity by targeting TGF-β signaling.

Antifibrosis drugs such as tranilast, pirfenidone, and losartan have shown encouraging efficacy in cancer treatment. Tranilast, for instance, has been demonstrated to reduce matrix mechanical pressure, lower tissue fluid hydrostatic pressure, and enhance tumor perfusion. And, it can enhance the efficacy of chemotherapy drugs with different molecular sizes, including doxorubicin, paclitaxel, and doxorubicin liposomes, by suppressing TGF-β signaling and expression of ECM components (63). Moreover, the combination of TMZ and tranilast significantly suppresses GBM patient-derived xenografts compared to TMZ alone (119, 120). Collectively, repurposing tranilast can not only enhance the efficacy of conventional chemotherapy drugs but also improve the effectiveness of antitumor nanomedicines. Similarly, pirfenidone, another antifibrosis drug that has been clinically approved for the treatment of idiopathic pulmonary fibrosis, also exhibits the function of reducing collagen and hyaluronic acid synthesis (33). Pirfenidone is confirmed to inhibit TGF-β expression in malignant glioma cells, indicating its further application as an adjunctive drug to sensitize glioma TMZ chemotherapy (121). Losartan (LOS), an angiotensin receptor blocker, can reduce the production of collagen and hyaluronic acid by downregulating profibrotic signals such as TGF-β1, CCN2, and ET-1 (70). Therefore, LOS may enhance chemotherapy efficacy by upregulating vascular perfusion and reducing the solid-phase pressure in tumors, which improves the delivery of drugs and oxygen to tumors. Additionally, LOS could antagonize the neoangiogenetic, profibrotic, and immunosuppressive effects of angiotensin II and significantly inhibit its stimulatory effects on local estrogen production, suppressing glioma cell growth and alleviating cerebral edema (122, 123). As a cost-effective angiotensin receptor blocker with an established safety profile, LOS can be quickly repurposed as an adjuvant pharmacological tool prospectively for GBM.

Recent studies have indicated that histone deacetylase inhibitors (HDACi) exhibit antifibrotic effects in various experimental models by preventing histone deacetylation, inducing chromatin decondensation and antifibrotic genes upregulation (124, 125). Valproic acid (VPA), an HDACi agent, exerts its antifibrotic effects by upregulating Smad7 and inhibiting the TGF-β/Smad signaling pathway (126). VPA has been found to inhibit fibrosis in experimental models of various diseases, including liver (127), kidney (128), and heart diseases (129), by reducing macrophage infiltration and downregulating the TGF-β signaling pathway. Briefly, VPA exhibits a dual-purpose effect in glioma therapy, as it not only functions as antiepileptics but also sensitizes TMZ chemotherapy in brain tumor patients (130–132). Another HDACi, vorinostat, approved by the U.S. FDA for the treatment of T-cell lymphoma (133), reduces collagen formation and inhibits fibrosis (134). Studies have shown that the combination of vorinostat and TMZ significantly enhances TMZ efficacy for glioma (135, 136). However, it remains unclear whether the enhanced chemosensitivity induced by VPA and vorinostat is partly or mainly modulated by the inhibition of glioma-associated fibrosis.

Interestingly, Chinese traditional medicine with antifibrosis properties also demonstrates its antitumor efficacy. Berberine, an isoquinoline alkaloid present in many traditional Chinese medicines (137), is confirmed to reduce collagen accumulation in pulmonary fibrosis (138), diabetic nephropathy (138), and arthritis (139), the related mechanisms of which may involve inhibiting TGF-β signaling (140) and restraining EMT (141). Moreover, berberine could suppress glioma growth, migration, and invasion by inhibiting COL11A1 expression and also induce programmed cell death through ERK1/2-mediated mitochondrial damage in glioma cells (142). These studies suggest that berberine could inhibit glioma growth possibly through its antifibrosis properties. Therefore, these conventionally approved antifibrosis drugs could be used to sensitize chemotherapy in glioma through inhibition of TGF-β signaling.

### 7.3 Targeting CAFs

Various antitumor strategies have been developed by directly targeting CAFs (74) including the depletion (73) and normalization of CAFs (143). As for certain cancers, the population of CAFs consists of a collection of multiple subsets of cells with diverse and specific phenotypes at different developmental stages. FAP, a universally acknowledged marker of CAFs, serves as a potential target in both antitumor and antifibrosis therapies. In glioma, FAP expression is detected in glioma cells, mesenchymal cells, and pericytes, etc. (144). Studies have developed an oncolytic adenovirus targeting both GBM cells and GBM-associated stromal FAP^+^ cells, highlighting its potential immunotherapy through depleting FAP^+^ CAFs (145). Additionally, FAP-targeting CAR‐T cells have demonstrated promising efficacy in a mouse xenograft model of GBM (146). Another new CAF phenotype in breast cancer, CD10^+^ GPR77^+^ CAFs, has been found to be associated with the acquisition of a chemoresistance phenotype. Targeting CD10^+^ GPR77^+^ CAFs has been demonstrated to retard tumor formation and reverse chemoresistance by destroying the survival niches for CSCs in both breast and lung cancers (25). However, apart from FAP^+^ CAFs, further research is needed to explore specific CAF phenotypes associated with glioma chemoresistance.

**Figure 3:**
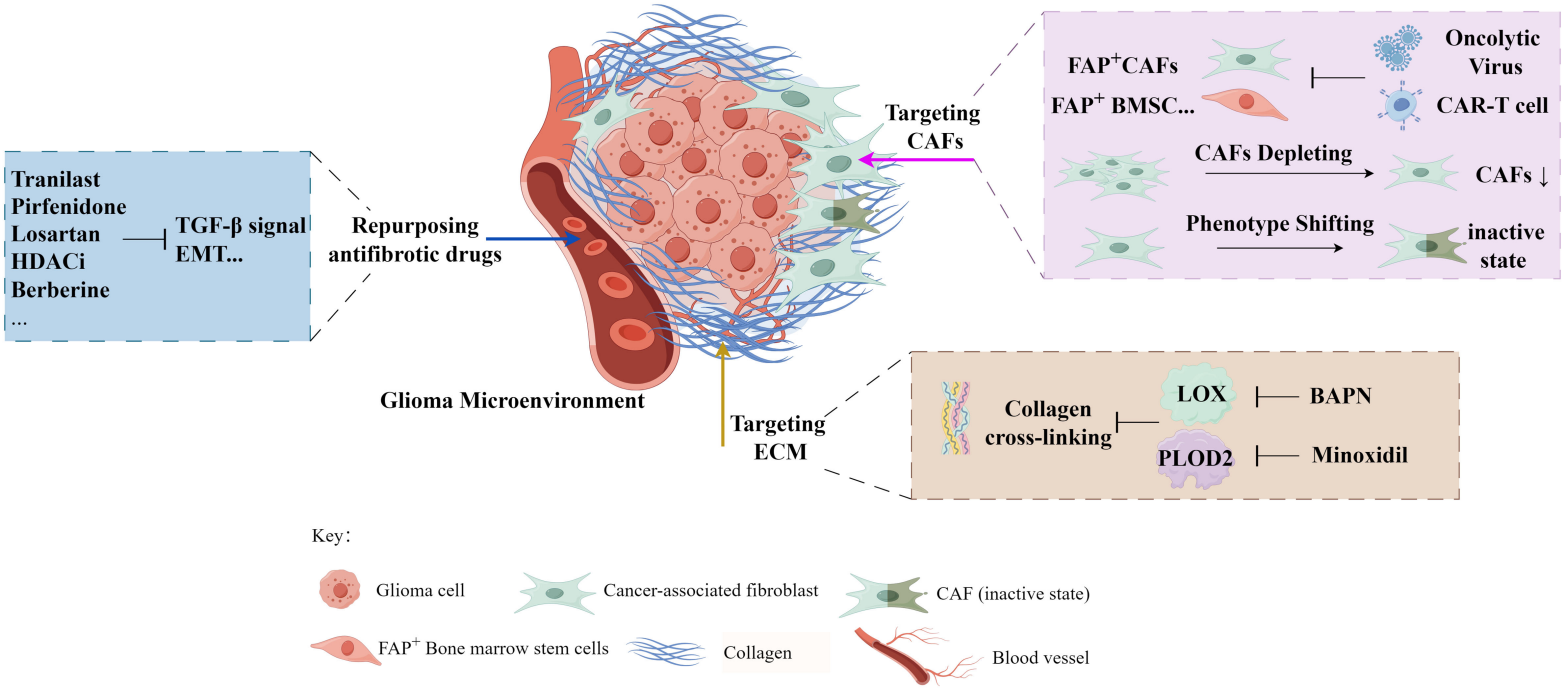
The exploration of antifibrosis-related strategies in glioma treatment (*by Figdraw*).

Above all, we summarized the current glioma therapies with different antifibrosis targets (**Figure 3**, **Table 1**).

**Table 1:** Drugs with antifibrosis function are utilized and tested in the treatment of solid tumors including glioblastoma.

Drug Name	Conditions	Highest Status (phase)	NCT	Status	Sample size
**Pirfenidone**	**Non-small cell lung cancer**	**II**	**NCT04467723**	**Recruiting**	**25**
**Tranilast**	**Nasopharyngeal carcinoma**	**II**	**NCT05626829**	**Recruiting**	**18**
**BAPN**	**Glioblastoma**	**Preclinical**	**-**	**-**	**-**
**Minoxidil**	**Ovarian cancer**	**II**	**NCT05272462**	**Recruiting**	**34**
**Losartan**	**Glioblastoma**	**Ⅲ**	**NCT01805453**	**Completed**	**80**
**Valproic acid**	**Glioblastoma**	**Ⅲ**	**NCT03243461**	**Recruiting**	**167**
**Vorinostat**	**High-grade glioma**	**Ⅲ**	**NCT01236560**	**Completed**	**101**
**Berberine**	**Non-small cell lung cancer**	**II**	**NCT03486496**	**Unknown**	**50**

## 8 Conclusion and future perspective

**Figure 4:**
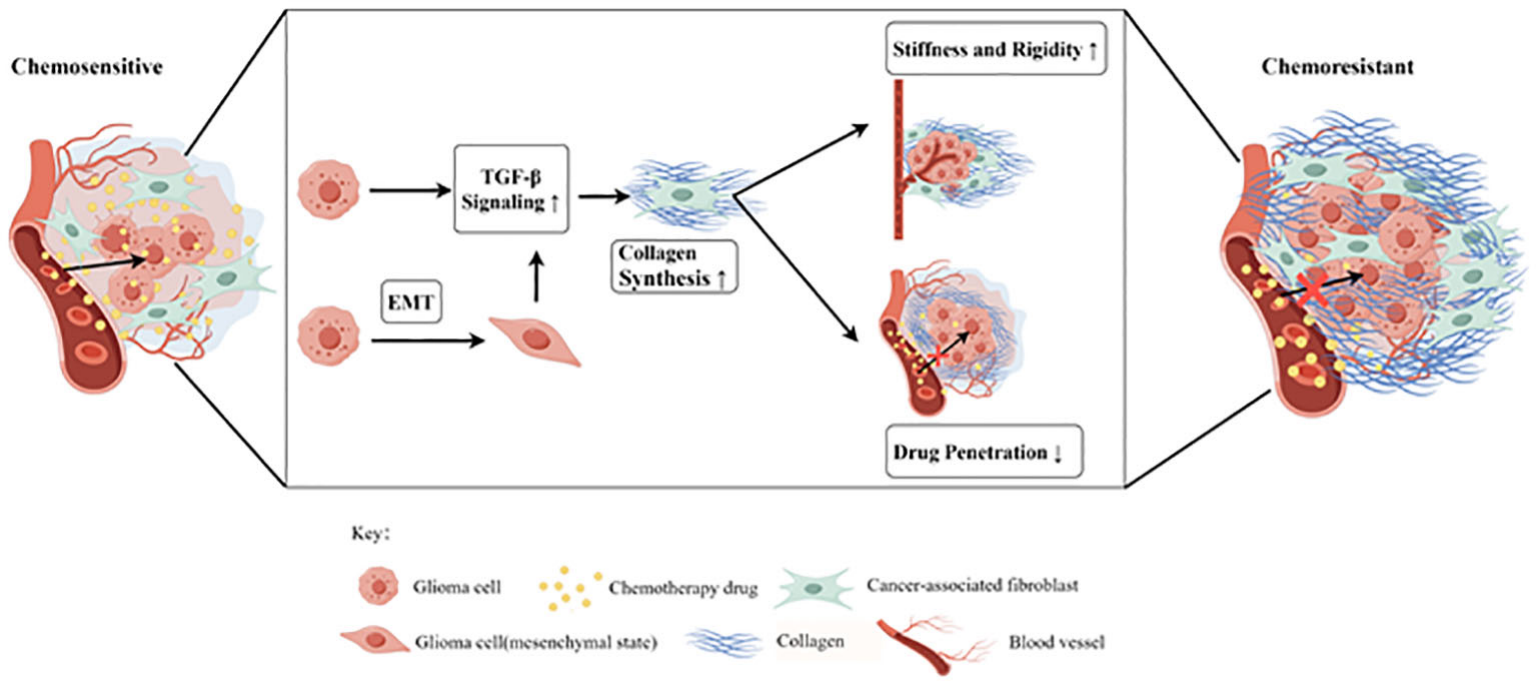
The tumor-associated fibrosis aggravates glioma chemoresistance by reducing the efficacy of drug delivery (*by Figdraw*). The TGF-β signaling pathway could be upregulated in glioma after chemotherapy and epithelial-mesenchymal transition (EMT), resulting in the increased collagen synthesis of cancer-associated fibroblasts (CAFs). In the tumor microenvironment (TME), the tumor-associated fibrosis increased the stiffness and rigidity of glioma tissues which in turn impairs the delivery of chemotherapeutic drugs to cancer cells, thus promoting chemoresistance.

Collectively, the mechanisms associated with glioma cells chemoresistance development can be attributed to two aspects: chemoresistance-related genetic alterations within glioma cells, and the GME changes contributing to drug resistance. The latter is, in part, induced and modulated by glioma-associated fibrosis, leading to increased tumor stiffness and decreased efficiency of chemotherapeutics delivery to the cancer cell nuclei. The features of the fibrotic GME include the abnormal vascular system, heightened ECM deposition, increased tumor stiffness, upregulated growth factors, etc. We further emphasize the crucial role of glioma-associated fibrotic reactions in glioma progression, prognosis, and chemoresistance. Intense glioma-associated fibrotic reactions positively correlate with poor outcomes in glioma patients, suggesting its clinical significance as both a prognostic indicator and a promising therapeutic target for overcoming glioma chemoresistance. Additionally, we propose a theory that chemotherapy-induced activation of TGF-β signaling could lead to tumor-associated fibrotic reactions in the GME, characterized by increased ECM stiffness. This, in turn, may hinder the penetration of chemotherapeutics into glioma cells. In this review, we emphasize that tumor-associated fibrotic reactions play a role in maintaining glioma stemness, leading to the acquisition of a chemoresistant phenotype (**Figure 4**). A comprehensive understanding of this mechanism promises new insights into effectively reversing chemoresistance. This review underscores the urgent need to decipher the complex relationship between glioma-associated fibrosis and chemotherapy sensitivity, providing a promising strategy to develop more effective interventions for glioma.

Despite our extensive summarization of numerous studies on how tumor-associated fibrosis facilitates chemoresistance, the exact molecular mechanisms still remain elusive in glioma. Therefore, in the future, it’s warranted to explore which molecular characteristics of glioma are more likely to develop fibrosis, and whether the ECM stiffness promotes the expression of chemoresistance-related proteins in glioma. Such insights would contribute to a deeper understanding of the interactions among these various chemoresistance mechanisms, potentially unveiling novel strategies to overcome chemoresistance. In addition to therapeutic agents directly targeting cancer cells, several innovative drugs are under investigation for their potential to overcome chemoresistance through modulating the TME. The antifibrosis therapy for solid tumors is one of the TME normalization strategies, with some showing significant tumor inhibition effects. Numerous studies have suggested that targeting CAFs and fibrosis with conventional clinically approved agents can enhance the chemosensitivity of solid tumors. However, further in-depth research is required to determine their efficacy specifically in the context of glioma treatment.

## Author contributions

JX: Writing – review & editing, Writing – original draft. JZ: Writing – review & editing. WC: Visualization, Writing – review & editing. XN: Writing – review & editing.
